# Bone markers and cardiovascular risk in type 2 diabetes patients

**DOI:** 10.1186/s12933-018-0691-2

**Published:** 2018-03-23

**Authors:** Sabine R. Zwakenberg, Yvonne T. van der Schouw, Casper G. Schalkwijk, Annemieke M. W. Spijkerman, Joline W. J. Beulens

**Affiliations:** 1 Julius Center for Health Sciences and Primary Care, University Medical Center Utrecht, PO box 85500, 3508 GA Utrecht, The Netherlands; 2 Department of Internal Medicine, Maastricht University Medical Centre, Maastricht, The Netherlands; 3 CARIM School for Cardiovascular Diseases, Maastricht University Medical Centre, Maastricht, The Netherlands; 4 Center for Nutrition, Prevention and Health Services, National Institute for Public Health and the Environment, Bilthoven, The Netherlands; 5 Department of Epidemiology & Biostatistics, EMGO+ Institute for Health and Care Research, VU University Medical Center, Amsterdam, The Netherlands

**Keywords:** Osteocalcin, Osteonectin, Osteopontin, Osteoprotegerin, Sclerostin, Alkaline phosphatase, Cardiovascular disease

## Abstract

**Background:**

Vascular calcifications are associated with a three- to fourfold increased risk of cardiovascular disease (CVD) and are highly prevalent in type 2 diabetes patients. Emerging evidence indicates that vascular calcification is a process of active bone formation regulated by stimulators and inhibitors of calcification. Therefore, this study aimed to evaluate whether six bone markers are associated with CVD risk in patients with type 2 diabetes.

**Methods:**

We used data of a case-cohort study, nested in the EPIC-NL cohort, comprising 134 CVD cases and a random subcohort of 218 participants, all with type 2 diabetes at baseline. Six bone markers (osteocalcin, osteopontin, osteonectin, osteoprotegerin, alkaline phosphatase and sclerostin) were measured in baseline plasma samples with multiplex assays and information on CVD events was obtained. The association of bone makers with CVD risk was evaluated using Cox proportional hazard analyses.

**Results:**

Higher concentrations of plasma osteopontin were associated (p_trend_ < 0.01) with an increased CVD risk with a hazard ratio of 2.00 (95%-CI 1.20–3.35) for the highest versus the lowest quartile in a multivariable adjusted model. The other bone markers were not associated with CVD risk.

**Conclusions:**

Higher osteopontin concentrations were associated with an increased CVD risk in type 2 diabetes patients. No consistent associations were found for the other five bone markers and risk of CVD in type 2 diabetes patients.

**Electronic supplementary material:**

The online version of this article (10.1186/s12933-018-0691-2) contains supplementary material, which is available to authorized users.

## Introduction

Patients with diabetes are at two- to fourfold increased risk of cardiovascular disease (CVD), which cannot be fully explained by traditional risk factors [1, 2]. Vascular calcification was long seen as a passive process of calcium deposition; however, recent evidence indicates it is an active process influenced by several stimulating and inhibiting factors [3]. Vascular calcification is a process with many similar mechanisms to bone formation, like the mineralization of vascular smooth muscle cells (VSMC) [4]. VSMC differentiate to osteoblast-like cells, due to exposure to a variety of stresses [5]. Expression of multiple bone markers has indeed been found in atherosclerotic plaques [6]. Moreover, hyperglycemia may induce VSMC proliferation, as well as the expression of some bone markers [7, 8].

Osteocalcin, osteopontin, and osteonectin have been suggested to inhibit mineralization of the VSMC by blocking hydroxyapatite formation [3]. Studies investigating the association between osteocalcin and CVD risk showed inconsistent results [9–11]. Osteopontin and osteonectin have been found in calcified vessels [6, 12, 13] and one study showed that osteocalcin and osteonectin may predict cardiovascular disease [14]. Several studies also showed that osteopontin may be a predictor of CVD risk in patients with type 2 diabetes [14–16]. However, prospective etiological evidence investigating the association between these bone markers and CVD events is lacking.

Osteoprotegerin, alkaline phosphatase, and sclerostin are suggested to play a role in CVD risk. Osteoprotegerin may inhibit osteoclast differentiation in bone and vascular tissue, and may therefore be considered as a protective factor for vascular calcification and CVD risk [3]. Alkaline phosphatase is suggested to cause an impaired vascular homeostasis and increase bone metabolism [17]. Subsequently, a recent meta-analysis showed that higher levels of alkaline phosphatase were associated with increased CVD risk [17]. Finally, sclerostin is a potent inhibitor of bone formation, but the available evidence with CVD risk is limited and inconclusive and mainly studied in patients with an impaired kidney function [18–22], since sclerostin levels increase with the progression of kidney function [23].

Presently, etiological studies investigating the association of these different bone markers and CVD incidence are far from conclusive and mainly studied in the general population or patients with chronic kidney disease. Furthermore, most bone markers are not studied prospectively, and none of the bone markers have been studied in patients with type 2 diabetes. Therefore, this study aims to evaluate whether six bone markers (osteocalcin, osteopontin, osteonectin, osteoprotegerin, alkaline phosphatase, sclerostin) are associated with CVD risk in patients with type 2 diabetes.

## Methods

### Study population

The EPIC-NL cohort is the Dutch contribution to the European Prospective Investigation into Cancer and Nutrition (EPIC) and consists of the Prospect-EPIC and MORGEN-EPIC cohorts [24]. In brief, the Prospect-EPIC study includes 17,357 women 49–70 years of age living in Utrecht and vicinity who participated in the nationwide Dutch breast cancer screening program. The MORGEN-EPIC cohort consists of 22,654 men and women 21–64 years of age selected from random samples of the Dutch population in three different towns. Participants were recruited in both studies from 1993 to 1997. Three sources of ascertainment of diabetes were used: self-report, hospital discharge diagnoses, and urinary strip test (in the Prospect part of the cohort only). Ascertained cases of diabetes were verified against medical and pharmacy records. Details of the ascertainment sources and verification procedures have previously been described [25].

Case-cohort sampling was performed to reduce costs and preserve valuable biological material. The case-cohort design is a method in which a random sample of the cohort at baseline, the subcohort, is used as control group for all cases that occur in the cohort [26]. From the 40,011 EPIC-NL participants, 526 participants were diagnosed with verified type 2 diabetes at baseline. During follow-up, 172 of the 526 participants with type 2 diabetes had a CVD event, of which 134 had blood samples available at baseline. These 134 participants were included as cases in our analyses. Additionally, a random sample of 218 participants from the diabetes participants was selected to serve as a subcohort in the case-cohort design. Because of the random sampling, this subcohort included 65 CVD cases (Additional file 1: Figure S1).

### Ethics, consent and permissions

All participants provided written informed consent before study inclusion. The study complies with the Declaration of Helsinki and was approved by the institutional board of the University Medical Center Utrecht (Prospect) and the Medical Ethical Committee of The Netherlands Organization for Applied Scientific Research Nutrition and Food Research (MORGEN).

### Bone markers

At baseline, we measured six different bone markers in plasma samples. All bone markers were measured using the human bone marker panel II multiplex assay (Meso Scale Discovery, Rockville MD, USA). Details of this multiplex assay, minimal detection limits and inter- and intra assay coefficients, are described in Additional file 1: Table S1.

### Clinical endpoints

Follow-up data on cardiovascular events were obtained from the Dutch Centre for Health Care Information, which holds a standardized computerized register of hospital discharge diagnoses. Follow-up was complete until the first of January 2008. Information on vital status was obtained through linkage with the municipal registries. Causes of death were collected from Statistics Netherlands, and coded according to the ICD-9 and ICD-10. Primary outcome for the present analysis was cardiovascular events (fatal and non-fatal), including coronary heart disease (CHD), congestive heart failure (CHF), peripheral arterial disease (PAD) and stroke. These clinical endpoints included both fatal and non-fatal CVD events. If a participant developed multiple cardiovascular events, the first event was taken into account in the analyses of total cardiovascular events. Since the mechanism behind the association between bone markers and CVD was mainly based on vascular calcification, CHD was analyzed separately. Due to limited statistical power, fatal events were analyzed separately only for total cardiovascular events.

### Other measurements

At baseline, participants completed general questionnaires about demographic and lifestyle characteristics and risk factors for chronic diseases. Smoking status was categorized as current, past, or never smoking. Physical activity was measured with the validated Cambridge Physical Activity Index, and was categorized as inactive, moderately inactive, moderately active and active [27].

Height and weight were measured by trained staff, and body mass index (BMI) was calculated as weight in kilograms divided by squared height in meters. Time since diabetes diagnosis was calculated by subtracting the age of diagnosis from the age at baseline examination and the estimated glomerular filtration rate (eGFR) was calculated based on creatinine with the CKD-EPI formula [28]. HbA1c was measured in erythrocytes using an immunoturbidimetric latex test. All measurements were performed according to standard operating procedures.

### Statistical analyses

Baseline characteristics were summarized as mean ± SD or median (IQR) as appropriate for continuous variables. In order to understand the possible confounding factors of the different bone markers, baseline characteristics were summarized in quartiles of the different bone markers. Furthermore, the correlation between the different bone markers was tested using the Spearman’s rank correlation coefficients. Since only for six persons data on eGFR was missing, we imputed these values using single imputation.

Cox proportional hazards models were used to estimate hazard ratio’s (HR) for each individual bone marker and cardiovascular events. Standard errors were calculated using the Prentice weighting method to account for the case-cohort design [29]. All subcohort members, both cases and non-cases, are weighted equally. Cases outside the subcohort are not weighted before the event. At the time of event, cases have the same weight as the subcohort members, and only then contribute to the risk set. We adjusted for risk factors of chronic diseases in three separate models. Age was the underlying time scale, with entry time defined as the participant’s age at recruitment and exit time as age at first fatal or non-fatal CVD event or censoring (whichever came first). The first model was adjusted for sex. The second model was additionally adjusted for risk factors for chronic diseases; BMI, smoking status (never/former/current), prevalent CVD, high sensitive C-reactive protein (hs-CRP), total- and HDL cholesterol. The third model was additionally adjusted for factors indicating the severity of diabetes; the age at onset of diabetes, HbA1c levels and eGFR. Analyses were performed in in quartiles and continuously, per 5 or 10 ng/ml, dependent on the scale of the bone markers. P-values for linear trends were calculated over the tertiles by entering the median value of each tertile as a linear covariate in the model.

Osteocalcin, osteopontin and osteonectin are all involved in blocking hydroxyapatite formation [3], and may complement each other. Therefore, a risk score was created; osteocalcin, osteopontin, and osteonectin were log transformed, a z-score was calculated for these individual bone markers and these values were summed up. A log-transformed score was used since the bone markers were not normally distributed.

Effect modification by sex was checked for all bone markers by including interaction terms in the model. In case of an interaction term with a P-value below 0.10, the analyses were stratified. In sensitivity analyses, we excluded the participants with prior CVD. Furthermore, CVD mortality and CHD incidence were analyzed separately. Because of a smaller sample in the sensitivity analyses, we analyzed these associations only continuously (per 5 or 10 ng/ml). All statistical analyses were performed using R 3.1.1 for Windows and a two-tailed P-value < 0.05 was considered statistically significant.

## Results

Baseline characteristics of the study population are shown in Table 1. In this study population, 82% were women, had an average age of 58 years, mean body mass index of 29.4 kg/m^2^ and a median duration of diagnosed diabetes of 5.3 years. Baseline characteristics of the subcohort according to the quartiles of the bone markers are shown in Additional file 1: Table S2. Osteocalcin and osteopontin levels were correlated (r = 0.58), and osteoprotegerin and osteopontin levels were moderately correlated (r = 0.28). The other bone markers were not correlated.

**Table 1:** Baseline characteristics for participants of the subcohort and cases

	Subcohort (n = 218)^a^	Cases (n = 134)
Age (years)	57.9 ± 6.7	58.6 ± 6.9
Women, n (%)	178 (82)	107 (80)
Diabetes duration (years)	5.3 (2.3–10.2)	6.3 (2.6–12.3)
History of CVD, n (%)	30 (14)	40 (30)
Current smoker, n (%)	49 (22)	42 (31)
BMI (kg/m^2^)	29.4 ± 4.9	30.3 ± 4.9
Systolic blood pressure (mmHg)	141.7 ± 20.8	145.6 ± 23.3
Total cholesterol (mmol/l)	5.2 ± 1.2	5.4 ± 1.1
HDL cholesterol (mmol/l)	1.0 ± 0.3	1.0 ± 0.3
HbA1c (%)	8.1 ± 1.7	8.2 ± 1.7
eGFR (ml/min/1.73 m^2^)	107.3 ± 24.1	109.1 ± 19.4
Hs-CRP (µg/mmol)	3.3 (1.8–6.5)	3.9 (2.5–6.6)
Osteocalcin (ng/ml)	22.6 (17.1–29.2)	21.7 (17.2–28.7)
Osteopontin (ng/ml)	12.8 (10.1–16.1)	13.5 (11.1–17.4)
Osteonectin (ng/ml)	114.6 (90.0–154.8)	124.4 (95.7–170.4)
Osteoprotegerin (ng/ml)	191.9 ± 55.6	201.0 ± 57.4
Alkaline Phosphatase (ng/ml)	55.8 (46.2–64.2)	56.2 (46.1–70.4)
Sclerostin (ng/ml)	60.3 (47.6–79.2)	60.8 (49.7–76.0)

Data are shown as frequencies (%), mean ± standard deviation or median (interquartile range)

*BMI* body mass index, *HDL* high density lipoprotein, *eGFR* estimated glomerular filtration, *Hs-CRP* high sensitive C-reactive protein

^a^This subcohort included 65 CVD cases

During the median survival time of 11 years, 134 participants experienced a CVD event (77 CHD, 19 stroke, 30 PAD, 8 CHF), of whom 46 fatal. The associations between the individual bone markers and CVD events are shown in Table 2. No association was observed for osteocalcin and osteonectin and CVD risk in the three different models with HR_Q4 vs Q1_ 1.17 (0.70–1.93) and HR_Q4 vs Q1_ 1.16 (0.72–1.88) for osteocalcin and osteonectin respectively. Higher concentrations of plasma osteopontin were associated with an increased CVD risk in the final models [model 3, HR_Q4 vs Q1_ 2.00 (1.20–3.35)], which was adjusted for sex, BMI, smoking status, prevalent CVD, hs-CRP, total cholesterol, HDL cholesterol, age at onset of diabetes, HbA1c levels and eGFR. A significant p for trend was observed in all models (p < 0.05). Similar results were observed when we analyzed osteopontin per 5 ng/ml increase with a HR of 1.11 (1.01–1.21), in the multivariable adjusted model (model 3). No associations were observed between osteoprotegerin [model 3, HR_Q4 vs Q1_ 1.23 (0.69–2.19)], alkaline phosphatase [model 3, HR_Q4 vs Q1_ 1.05 (0.60–1.85)] and sclerostin [model 3, HR_Q4 vs Q1_ 0.75 (0.41–1.36)] and CVD risk. Osteonectin, osteocalcin, and osteopontin were combined to one bone marker-score. Increased levels of this score were associated with an increased CVD risk [model 3, HR_Q4 vs Q1_ 1.66 (1.03–2.62)].

**Table 2:** The HR for each individual bone marker and the effect on CVD

	Q1	Q2	Q3	Q4	P_trend_	Continuous
Osteocalcin						
(Median, ng/ml)	14.22	19.68	25.45	34.75		
Events	33/68	41/80	29/67	31/73		Per 5 ng/ml
Model 1	Ref	0.91 (0.58–1.42)	0.74 (0.44–1.25)	0.83 (0.51–1.35)	0.40	1.01 (0.98–1.02)
Model 2	Ref	0.90 (0.59–1.47)	0.94 (0.55–1.60)	1.10 (0.67–1.81)	0.64	1.01 (0.99–1.02)
Model 3	Ref	0.83 (0.51–1.36)	0.92 (0.53–1.59)	1.17 (0.70–1.93)	0.43	1.01 (1.00–1.03)
Osteopontin						
(Median, ng/ml)	8.84	11.66	13.78	18.72		
Events	23/63	31/70	36/74	44/81		Per 5 ng/ml
Model 1	Ref	1.18 (0.70–1.97)	1.51 (0.89–2.56)	1.70 (1.03–2.78)	0.02	1.08 (0.97–1.20)
Model 2	Ref	1.34 (0.78–2.33)	1.79 (1.06–3.02)	1.76 (1.08–2.88)	0.02	1.08 (0.98–1.20)
Model 3	Ref	1.36 (0.76–2.44)	1.66 (0.95–2.89)	2.00 (1.20–3.35)	< 0.01	1.11 (1.01–1.21)
Osteonectin						
(Median, ng/ml)	71.78	103.40	127.9	195.6		
Events	29/70	23/65	41/75	41/78		Per 10 ng/ml
Model 1	Ref	0.83 (0.48–1.44)	1.63 (1.02–2.60)	1.39 (0.85–2.20)	0.08	1.02 (1.00–1.03)
Model 2	Ref	0.77 (0.44–1.32)	1.32 (0.82–2.14)	1.16 (0.72–1.88)	0.30	1.02 (1.00–1.04)
Model 3	Ref	0.74 (0.43–1.27)	1.25 (0.76–2.05)	1.16 (0.72–1.88)	0.30	1.02 (1.00–1.04)
Osteoprotegerin						
(Median, ng/ml)	134.4	168.3	199.0	256.6		
Events	30/69	28/69	32/73	44/77		Per 10 ng/ml
Model 1	Ref	0.88 (0.53–1.46)	0.91 (0.56–1.46)	1.51 (0.91–2.52)	0.07	1.03 (1.00–1.06)
Model 2	Ref	0.82 (0.50–1.36)	0.87 (0.53–1.44)	1.13 (0.65–1.98)	0.46	1.01 (0.98–1.04)
Model 3	Ref	0.88 (0.52–1.47)	0.84 (0.49–1.45)	1.23 (0.69–2.19)	0.35	1.01 (0.98–1.05)
Alkaline phosphatase						
(Median, ng/ml)	42.0	51.39	59.7	74.4		
Events	35/68	31/73	25/68	43/79		Per 10 ng/ml
Model 1	Ref	0.79 (0.49–1.29)	0.67 (0.40–1.14)	1.13 (0.71–1.79)	0.52	1.07 (0.94–1.21)
Model 2	Ref	0.74 (0.45–1.22)	0.67 (0.39–1.16)	1.03 (0.61–1.73)	0.64	1.02 (0.89–1.18)
Model 3	Ref	0.79 (0.47–1.35)	0.68 (0.39–1.17)	1.05 (0.60–1.85)	0.66	1.03 (0.89–1.19)
Sclerostin						
(Median, ng/ml)	40.65	53.6	67.8	93.6		
Events	29/65	35/73	42/79	28/71		Per 10 ng/ml
Model 1	Ref	1.07 (0.65–1.78)	1.18 (0.74–1.89)	0.83 (0.49–1.40)	0.42	0.99 (0.97–1.02)
Model 2	Ref	1.05 (0.64–1.73)	1.04 (0.64–1.67)	0.76 (0.44–1.32)	0.27	0.99 (0.96–1.02)
Model 3	Ref	1.15 (0.68–1.93)	1.07 (0.65–1.75)	0.75 (0.41–1.36)	0.24	0.99 (0.96–1.02)

Model 1: Adjusted of sex (age is the underlying time scale)

Model 2: Additionally adjusted for BMI, smoking status, prevalent CVD, hs-CRP, total cholesterol and HDL cholesterol

Model 3: Additionally adjusted for age at onset of diabetes, HbA1c levels and eGFR

### Sensitivity analyses

The associations between the different bone markers and CHD incidence (n = 77) and CVD mortality (n = 46) were similar to those with CVD incidence. The hazard ratio (model 3) for the association between osteopontin and CHD and CVD mortality was HR_per 5 ng/ml_ 1.11 (0.97–1.28) and HR_per 5 ng/ml_ 1.13 (0.95–1.33) for CHD incidence and CVD mortality respectively. Excluding participants with a prior CVD event resulted in 237 participants for analyses in whom 94 CVD events occurred during follow up, whereof 45 events occurred in the subcohort. The Hazard Ratio slightly dropped to unity, and no significant association was observed [HR_per 5 ng/ml_ 1.08 (0.97–1.19)]. The associations of the other bone markers and CVD events remained similar. No significant interaction was present between any bone markers and sex.

## Discussion

The aim of this study was to assess the association between multiple bone markers and CVD risk in patients with type 2 diabetes. We did not observe any consistent associations for bone markers and risk of CVD. Only elevated osteopontin levels were associated with higher CVD risk.

To the best of our knowledge, our study is the first prospective cohort study investigating associations between a variety of bone markers and CVD risk in patients with type 2 diabetes. Nevertheless, some limitations need to be addressed. This study was underpowered to explore the association between these multiple bone markers and specific CVD endpoints such as stroke, PAD and CHF. The sensitivity analyses focusing on CVD mortality, CHD events and in participants without a prior CVD events seem to be underpowered. Additionally, including data on vascular calcification, like coronary artery calcium score, would provide more insights on the underlying mechanism of the association between bone markers and CVD risk.

We hypothesized that multiple bone markers were associated with CVD risk in patients with type 2 diabetes; however, we did not detect an association between these bone markers and CVD risk, except for osteopontin. The available literature is limited and inconsistent, especially cohort studies including diabetes patients are lacking. The evidence linking osteocalcin to CVD events showed contradicting results, showing an inverse association [11], null result [10] and positive association [9, 11]. Those studies reporting an association between osteocalcin and CVD risk, only detected an association in elderly (> 70 year); however, these associations were in opposite directions [9, 11]. Additionally, one previous study indicated that osteocalcin may be a marker of vascular disease in patients with type 1 diabetes [30]. Osteonectin was detected in atherosclerotic plaques, but cohort studies focusing on osteonectin and incident CVD risk are lacking, although one study suggested that osteonectin may predict CVD events [14]. The bone markers-score, including osteocalcin, osteonectin and osteopontin, was associated with CVD incidence, but this is mainly driven by osteopontin.

Based on previous meta-analyses, we mainly expected to find an association between osteoprotegerin, alkaline phosphatase and CVD risk [17, 31]. A recent meta-analysis showed that elevated osteoprotegerin concentration was associated with increased CVD risk in the general population [31]. Another study showed increased osteoprotegerin levels in patients with PAD compared to patients without PAD in participants with type 2 diabetes [32]. We did not detect an association between osteoprotegerin and CVD risk, but we were not able to investigate PAD incidence separately. The meta-analysis focusing on alkaline phosphatase included four prospective studies [33–36], of which two studies found an increased CVD risk [34, 36]. These studies focused on CVD risk in men [34] and stroke incidence [36] and found relative risks comparable to this present study, but the previous studies found significant associations. However, two other large scale prospective studies, focusing on stroke incidence and CVD mortality, did not find any association [33, 35]. The previous cohort studies investigating the association between alkaline phosphatase and CVD risk were all large-scale studies, and our study could be underpowered to detect an association [33–36].

Sclerostin levels increases with the progression of kidney disease, and is potent inhibitor of bone formation, and therefore suggested to reduce vascular calcification [22]. Sclerostin is only studied in patients with chronic kidney disease, and the cohort studies showed conflicting results which could be explained by small sample sizes and heterogeneity of the study population [22]. This study did not detect an association between sclerostin and CVD incidence in patients with type 2 diabetes; however, additional research is necessary to confirm this null result.

The current study is the first prospective study addressing the association between osteopontin and CVD events in patients with type 2 diabetes, and showed that higher osteopontin levels were associated with an increased CVD risk. This association is opposite to what is expected based on osteopontin’s function as an inhibitor of calcification. No significant association was present when investigating CHD specific incidence and CVD mortality, and suggests a lack of power (CHD incidence, n = 77, fatal CVD n = 46). Excluding participants with a prior CVD event resulted in a lower, non-significant HR, which also could be a power issue (CVD incidence, n = 94) or osteopontin might be upregulated due to calcification. Multiple studies suggested that osteopontin levels are elevated because of atherosclerosis [37, 38]. A study using coronary angiography showed that osteopontin levels were indeed associated with the presence and extent of coronary artery disease [38]. In line with our study, multiple studies also showed that elevated osteopontin may be a predictor of increased CVD risk in patients with diabetes [14–16], and osteopontin is elevated in heart failure patients [39, 40]. Furthermore, osteopontin may also be involved in the coronary calcifications process by inhibiting hydroxyapatite formation [3]. To this end, it has to be elucidated whether osteopontin is a marker of calcification, a protector or an active player in the calcification process; therefore, additional research is necessary to detect osteopontin’s role in the process of vascular calcification.

## Conclusion

No consistent associations were found for bone markers and risk of CVD in type 2 diabetes patients. Only higher osteopontin concentrations were associated with an increased CVD risk in type 2 diabetes patients. This association attenuated to the null after exclusion of the patients with a prior CVD event.

## Additional file

**Additional file 1.** Additional tables and figures.

## References

[1] Shaw JE, Sicree RA, Zimmet PZ (2010). Global estimates of the prevalence of diabetes for 2010 and 2030. Diabetes Res Clin Pract.

[2] The Emerging Risk Factors Collaboration (2010). Diabetes mellitus, fasting blood glucose concentration, and risk of vascular disease: a collaborative meta-analysis of 102 prospective studies. Lancet.

[3] Evrard S, Delanaye P, Kamel S, Cristol JP, Cavalier E, Arnaud J (2014). Vascular calcification: from pathophysiology to biomarkers. Clin Chim Acta..

[4] Demer LL, Tintut Y (2008). Vascular calcification: pathobiology of a multifaceted disease. Circulation..

[5] Leopold JA (2015). Vascular calcification: mechanisms of vascular smooth muscle cell calcification. Trends Cardiovasc Med..

[6] Bini A, Mann KG, Kudryk BJ, Schoen FJ (1999). Noncollagenous bone matrix proteins, calcification, and thrombosis in carotid artery atherosclerosis. Arterioscler Thromb Vasc Biol..

[7] Sun J, Xu Y, Dai Z, Sun Y (2009). Intermittent high glucose enhances proliferation of vascular smooth muscle cells by upregulating osteopontin. Mol Cell Endocrinol.

[8] Faries PL, Rohan DI, Takahara H, Wyers MC, Contreras MA, Quist WC (2001). Human vascular smooth muscle cells of diabetic origin exhibit increased proliferation, adhesion, and migration. J Vasc Surg..

[9] Yeap BB, Chubb SAP, Flicker L, McCaul KA, Ebeling PR, Hankey GJ (2012). Associations of total osteocalcin with all-cause and cardiovascular mortality in older men: the health in men study. Osteoporos Int.

[10] Hwang Y-C, Kang M, Cho I-J, Jeong I-K, Ahn KJ, Chung HY (2015). Association between the circulating total osteocalcin level and the development of cardiovascular disease in middle-aged men: a mean 8.7-year longitudinal follow-up study. J Atheroscler Thromb..

[11] Holvik K, van Schoor NM, Eekhoff EMW, den Heijer M, Deeg DJH, Lips P (2014). Plasma osteocalcin levels as a predictor of cardiovascular disease in older men and women: a population-based cohort study. Eur J Endocrinol.

[12] Mohamadpour AH, Abdolrahmani L, Mirzaei H, Sahebkar A, Moohebati M, Ghorbani M (2015). Serum osteopontin concentrations in relation to coronary artery disease. Arch Med Res.

[13] Singh M, Ananthula S, Milhorn DM, Krishnaswamy G, Singh K (2007). Osteopontin: a novel inflammatory mediator of cardiovascular disease. Front Biosci..

[14] van der Leeuw J, Beulens JWJ, van Dieren S, Schalkwijk CG, Glatz JFC, Hofker MH (2016). Novel biomarkers to improve the prediction of cardiovascular event risk in type 2 diabetes mellitus. J Am Heart Assoc..

[15] Berezin AE, Kremzer AA (2013). Circulating osteopontin as a marker of early coronary vascular calcification in type two diabetes mellitus patients with known asymptomatic coronary artery disease. Atherosclerosis..

[16] Gordin D, Forsblom C, Panduru NM, Thomas MC, Bjerre M, Soro-Paavonen A (2014). Osteopontin is a strong predictor of incipient diabetic nephropathy, cardiovascular disease, and all-cause mortality in patients with type 1 diabetes. Diabetes Care.

[17] Kunutsor SK, Apekey TA, Khan H (2014). Liver enzymes and risk of cardiovascular disease in the general population: a meta-analysis of prospective cohort studies. Atherosclerosis..

[18] Drechsler C, Evenepoel P, Vervloet MG, Wanne C, Ketteler M, Marx N (2015). High levels of circulating sclerostin are associated with better cardiovascular survival in incident dialysis patients: results from the NECOSAD study. Nephrol Dial Transplant.

[19] Viaene L, Behets GJ, Claes K, Meijers B, Blocki F, Brandenburg V (2013). Sclerostin: another bone-related protein related to all-cause mortality in haemodialysis?. Nephrol Dial Transplant.

[20] Morena M, Jaussent I, Dupuy A-M, Bargnoux A-S, Kuster N, Chenine L (2015). Osteoprotegerin and sclerostin in chronic kidney disease prior to dialysis: potential partners in vascular calcifications. Nephrol Dial Transplant.

[21] Kuipers AL, Miljkovic I, Carr JJ, Terry JG, Nestlerode CS, Ge Y (2015). Association of circulating sclerostin with vascular calcification in Afro-Caribbean men. Atherosclerosis..

[22] Kanbay M, Solak Y, Siriopol D, Aslan G, Afsar B, Yazici D (2016). Sclerostin, cardiovascular disease and mortality: a systematic review and meta-analysis. Int Urol Nephrol.

[23] Cejka D, Marculescu R, Kozakowski N, Plischke M, Reiter T, Gessl A (2014). Renal elimination of sclerostin increases with declining kidney function. J Clin Endocrinol Metab.

[24] Beulens JWJ, Monninkhof EM, Verschuren WMM, van der Schouw YT, Smit J, Ocke MC (2010). Cohort profile: the EPIC-NL study. Int J Epidemiol.

[25] Sluijs I, Beulens JWJ, Spijkerman AMW, Ros MM, Grobbee DE (2010). Ascertainment and verification of diabetes in the EPIC-NL study. Neth J Med.

[26] Onland-Moret NC, van der Schouw YT, Buschers W, Elias SG, van Gils CH (2007). Analysis of case-cohort data: a comparison of different methods. J Clin Epidemiol.

[27] Wareham NJ, Jakes RW, Rennie KL, Schuit J, Mitchell J, Hennings S (2003). Validity and repeatability of a simple index derived from the short physical activity questionnaire used in the European Prospective Investigation into Cancer and Nutrition (EPIC) study. Public Health Nutr..

[28] Inal BB, Oguz O, Emre T, Usta M, Inal H, Altunoglu E (2014). Evaluation of MDRD, Cockcroft–Gault, and CKD-EPI formulas in the estimated glomerular filtration rate. Clin Lab..

[29] Barlow WE, Ichikawa L, Rosner D, Izumi S (1999). Analysis of case-cohort designs. J Clin Epidemiol.

[30] Maddaloni E, Xia Y, Park K, D’Eon S, Tinsley LJ, St-Louis R (2017). High density lipoprotein modulates osteocalcin expression in circulating monocytes: a potential protective mechanism for cardiovascular disease in type 1 diabetes. Cardiovasc Diabetol.

[31] Tschiderer L, Willeit J, Schett G, Kiechl S, Willeit P (2017). Osteoprotegerin concentration and risk of cardiovascular outcomes in nine general population studies: literature-based meta-analysis involving 26,442 participants. PLoS ONE..

[32] Giovannini S, Tinelli G, Biscetti F, Straface G, Angelini F, Pitocco D (2017). Serum high mobility group box-1 and osteoprotegerin levels are associated with peripheral arterial disease and critical limb ischemia in type 2 diabetic subjects. Cardiovasc Diabetol.

[33] Shimizu Y, Imano H, Ohira T, Kitamura A, Kiyama M, Okada T (2013). Alkaline phosphatase and risk of stroke among Japanese: the circulatory risk in communities study (CIRCS). J Stroke Cerebrovasc Dis..

[34] Wannamethee SG, Sattar N, Papcosta O, Lennon L, Whincup PH (2013). Alkaline phosphatase, serum phosphate, and incident cardiovascular disease and total mortality in older men. Arterioscler Thromb Vasc Biol.

[35] Tonelli M, Curhan G, Pfeffer M, Sacks F, Thadhani R, Melamed ML (2009). Relation between alkaline phosphatase, serum phosphate, and all-cause or cardiovascular mortality. Circulation.

[36] Wieberdink RG, Koudstaal PJ, Hofman A (2011). Serum liver enzymes and the risk of stroke in the general population: the rotterdam study. Cardiovasc Dis.

[37] Wolak T (2014). Osteopontin—a multi-modal marker and mediator in atherosclerotic vascular disease. Atherosclerosis..

[38] Ohmori R, Momiyama Y, Taniguchi H, Takahashi R, Kusuhara M, Nakamura H (2003). Plasma osteopontin levels are associated with the presence and extent of coronary artery disease. Atherosclerosis..

[39] Rosenberg M, Zugck C, Nelles M, Juenger C, Frank D, Remppis A (2008). Osteopontin, a new prognostic biomarker in patients with chronic heart failure. Circ Heart Fail..

[40] Podzimkova J, Palecek T, Kuchynka P, Marek J, Danek BA, Jachymova M (2017). Plasma osteopontin levels in patients with dilated and hypertrophic cardiomyopathy. Herz..

