# Supplementary material for: Bone markers and cardiovascular risk in type 2 diabetes patients
Source: Cardiovasc Diabetol. 2018 Mar 23;17:45. doi: 10.1186/s12933-018-0691-2 (PMC5866514; doi:10.1186/s12933-018-0691-2)
Supplement: Supplementary file 1 — Additional file 1. Additional tables and figures. [file 12933_2018_691_MOESM1_ESM.docx]

| **Supplementary table 1.** Details of the multiplex assay, the minimal detection limit, inter- and intra assay variation coefficients. | | | |
| --- | --- | --- | --- |
|  | Detection limit (ng/ml) | Inter-assay variation coefficients (%) | Intra-assay variation coefficients (%) |
| Osteocalcin | 0.011 | 6.6 | 3.4 |
| Osteopontin | 0.027 | 8.0 | 4.0 |
| Osteonectin | 2.5 | 6.8 | 9.9 |
| Osteoprotegerin | 0.005 | 4.9 | 4.1 |
| Alkaline Phosphatase | 8.3 | 5.7 | 2.2 |
| Sclerostin | 0.003 | 5.6 | 5.4 |

| **Supplementary table 2.** Baseline characteristics by quartiles of the six bone markers in the subcohort (n=218). | | | | | |
| --- | --- | --- | --- | --- | --- |
| **Osteocalcin** | **Q1** | **Q2** | **Q3** | **Q4** | **Trend-test** |
| Age (years) | 56.5 ± 6.9 | 56.9 ± 6.9 | 59.5 ± 6.3 | 58.9 ± 6.2 | 0.05 |
| Women, n(%) | 45 (82) | 42 (78) | 42 (78) | 49 (89) | 0.38 |
| Diabetes duration (years) | 5.8 (2.7-8.1) | 5.5 (1.7-11.9) | 3.8 (2.5-14.8) | 5.2 (2.4-10.1) | 0.99 |
| History of CVD, n(%) | 9 (16) | 9 (17) | 8 (15) | 4 (7) | 0.44 |
| BMI (kg/m2) | 30.9 ± 5.1 | 29.6 ± 4.4 | 28.3 ± 4.7 | 28.8 ± 4.9 | 0.03 |
| Systolic BP (mmHg) | 141.0 ± 19.2 | 139.0 ± 17.8 | 141.6 ± 20.3 | 145.3 ± 25.2 | 0.45 |
| Current smoker, n(%) | 12 (22) | 19 (35) | 10 (19) | 8 (15) | 0.19 |
| Total cholesterol | 5.1 ± 1.2 | 5.3 ± 1.2 | 5.3 ± 1.2 | 5.1 ± 1.3 | 0.48 |
| HDL | 1.0 ± 0.3 | 1.0 ± 0.3 | 1.1 ± 0.3 | 1.1 ± 0.3 | 0.41 |
| HbA1c (%) | 8.4 ± 1.9 | 8.1 ± 1.7 | 7.8 ± 1.6 | 8.0 ± 1.4 | 0.38 |
| eGFR | 104.3 ± 14.3 | 106.3 ± 21.8 | 110.2 ± 29.4 | 108.4 ± 28.2 | 0.61 |
| CRP | 4.8 (2.7-7.8) | 3.3 (1.7-6.3) | 2.9 (1.4-5.0) | 2.8 (1.5-6.1) | 0.07 |
| **Osteopontin** | **Q1** | **Q2** | **Q3** | **Q4** |  |
| Age (years) | 56.3 ± 6.8 | 58.0 ± 5.1 | 58.5 ± 8.6 | 59.0 ± 5.5 | 0.16 |
| Women, n(%) | 44 (80) | 44 (82) | 44 (82) | 46 (84) | 0.97 |
| Diabetes duration (years) | 3.6 (1.7–8.0) | 5.9 (2.9-11.0) | 6.9 (3.2-14.3) | 3.8 (2.2-7.2) | 0.51 |
| History of CVD, n(%) | 10 (18) | 6 (11) | 7 (13) | 7 (13) | 0.73 |
| BMI (kg/m2) | 30.0 ± 5.2 | 29.8 ± 4.2 | 28.9 ± 5.2 | 28.9 ± 4.8 | 0.51 |
| Systolic BP (mmHg) | 140.2 ± 21.3 | 139.8 ± 16.8 | 143.9 ± 22.8 | 143.0 ± 22.1 | 0.67 |
| Current smoker, n(%) | 10 (18) | 18 (33) | 10 (19) | 11 (20) | 0.15 |
| Total cholesterol | 5.3 ± 1.2 | 5.1 ± 1.3 | 5.3 ± 1.0 | 5.1 ± 1.1 | 0.76 |
| HDL | 1.1 ± 0.3 | 1.1 ± 0.4 | 1.0 ± 0.3 | 1.0 ± 0.3 | 0.63 |
| HbA1c (%) | 8.3 ± 1.9 | 8.1 ± 1.7 | 8.1 ± 1.6 | 7.8 ± 1.5 | 0.62 |
| eGFR | 106.0 ± 19.1 | 101.6 ± 16.5 | 109.4 ± 22.7 | 112.1 ± 33.7 | 0.13 |
| CRP | 3.0 (1.9-6.4) | 3.5 (1.9-6.6) | 3.3 (1.5-5.6) | 4.0 (2.0-6.8) | 0.82 |
| **Osteonectin** | **Q1** | **Q2** | **Q3** | **Q4** |  |
| Age (years) | 58.6 ± 6.2 | 57.8 ± 5.6 | 56.4 ± 8.0 | 59.0 ± 6.3 | 0.18 |
| Women, n(%) | 49 (89) | 44 (82) | 43 (80) | 42 (76) | 0.36 |
| Diabetes duration (years) | 6.2 (2.6-9.7) | 6.4 (3.7-11.2) | 4.1 (2.5-10.5) | 3.1 (1.4-8.5) | 0.14 |
| History of CVD, n(%) | 10 (18) | 5 (9) | 10 (19) | 5 (9) | 0.28 |
| BMI (kg/m2) | 28.9 ± 4.4 | 29.0 ± 5.0 | 29.2 ± 4.9 | 30.4 ± 5.0 | 0.33 |
| Systolic BP (mmHg) | 142.8 ± 21.5 | 138.6 ± 18.6 | 138.7 ± 21.8 | 146.7 ± 20.7 | 0.13 |
| Current smoker, n(%) | 9 (16) | 14 (26) | 14 (26) | 12 (22) | 0.90 |
| Total cholesterol | 5.0 ± 1.2 | 5.2 ± 1.1 | 5.3 ± 1.1 | 5.4 ± 1.3 | 0.30 |
| HDL | 1.1 ± 0.4 | 1.1 ± 0.3 | 1.0 ± 0.2 | 1.0 ± 0.3 | 0.39 |
| HbA1c (%) | 7.9 ± 1.4 | 8.0 ± 1.7 | 8.0 ± 1.9 | 8.4 ± 1.7 | 0.45 |
| eGFR - MDRD | 108.6 ± 31.3 | 104.8 ± 14.3 | 106.4 ± 24.1 | 109.3 ± 24.3 | 0.75 |
| CRP | 2.7 (1.3-5.7) | 3.1 (1.7-6.6) | 4.0 (2.1-6.5) | 4.2 (2.2-7.0) | 0.25 |
| Data are shown as frequencies (%), mean ± standard deviation or median (interquartile range). BMI = body mass index, eGFR = estimated glomerular filtration rate. Linear trend was tested with ANOVA or Chi-square test, as appropriate. Skewed variables (diabetes duration and CRP) were loge-transformed prior to analyses. | | | | | |

| **Supplementary table 2 (continued).** Baseline characteristics by quartiles of the six bone markers in the  subcohort (n=218). | | | | | | | | | |
| --- | --- | --- | --- | --- | --- | --- | --- | --- | --- |
| **Osteoprotegerin** | | **Q1** | | **Q2** | | **Q3** | | **Q4** | **Trend-test** |
| Age (years) | | 54.6 ± 6.5 | | 56.0 ± 6.1 | | 59.2 ± 6.1 | | 62.0 ± 5.4 | <0.01 |
| Women, n(%) | | 40 (73) | | 42 (78) | | 46 (85) | | 50 (91) | 0.07 |
| Diabetes duration (years) | | 5.8 (2.2-10.5) | | 5.8 (2.2-8.7) | | 5.3 (1.8-10.2) | | 3.7 (2.6-11.2) | 0.91 |
| History of CVD, n(%) | | 4 (7) | | 7 (13) | | 6 (11) | | 13 (24) | 0.08 |
| BMI (kg/m2) | | 29.7 ± 4.7 | | 28.4 ± 4.1 | | 30.3 ± 5.5 | | 29.2 ± 5.0 | 0.24 |
| Systolic BP (mmHg) | | 135.8 ± 18.7 | | 138.9 ± 20.9 | | 142.2 ± 18.2 | | 150.0 ± 22.8 | <0.01 |
| Current smoker, n(%) | | 17 (31) | | 19 (35) | | 8 (15) | | 5 (9) | 0.01 |
| Total cholesterol | | 5.5 ± 1.1 | | 5.3 ± 1.2 | | 5.0 ± 1.1 | | 4.9 ± 1.2 | 0.03 |
| HDL | | 1.1 ± 0.3 | | 1.0 ± 0.3 | | 1.1 ± 0.3 | | 1.0 ± 0.3 | 0.07 |
| HbA1c (%) | | 7.8 ± 1.6 | | 7.6 ± 1.3 | | 8.4 ± 1.7 | | 8.4 ± 1.9 | 0.01 |
| eGFR | | 105.2 ± 13.8 | | 106.8 ± 30.7 | | 109.8 ± 27.3 | | 107.5 ± 21.7 | 0.80 |
| CRP | | 3.0 (1.4-5.6) | | 2.9 (1.3-6.9) | | 3.1 (1.8-6.2) | | 4.2 (2.3-6.9) | 0.16 |
| **Alkaline Phosphatase** | | **Q1** | | **Q2** | | **Q3** | | **Q4** |  |
| Age (years) | | 56.9 ± 6.7 | | 57.1± 7.3 | | 58.9 ± 6.1 | | 58.9 ± 6.3 | 0.21 |
| Women, n(%) | | 41 (75) | | 43 (80) | | 47 (87) | | 47 (86) | 0.31 |
| Diabetes duration (years) | | 6.9 (2.8-11.9) | | 4.9 (2.7-8.1) | | 5.3 (1.9-11.9) | | 4.2 (2.2-9.9) | 0.27 |
| History of CVD, n(%) | | 7 (13) | | 8 (15) | | 9 (17) | | 6 (11) | 0.84 |
| BMI (kg/m2) | | 29.0 ± 4.5 | | 29.7 ± 4.9 | | 29.5 ± 5.0 | | 29.4 ± 5.2 | 0.92 |
| Systolic BP (mmHg) | | 142.7 ± 19.0 | | 137.2 ± 19.4 | | 142.1 ± 21.3 | | 144.9 ± 23.2 | 0.26 |
| Current smoker, n(%) | | 13 (24) | | 11 (20) | | 11 (20) | | 14 (25) | 0.97 |
| Total cholesterol | | 5.1 ± 1.2 | | 5.2 ± 0.9 | | 5.4 ± 1.4 | | 5.1 ± 1.2 | 0.43 |
| HDL | | 1.1 ± 0.3 | | 1.1 ± 0.3 | | 1.0 ± 0.2 | | 1.0 ± 0.3 | 0.04 |
| HbA1c (%) | | 7.6 ± 1.2 | | 8.1 ± 1.8 | | 8.1 ± 1.7 | | 8.5 ± 1.9 | 0.04 |
| eGFR | | 109.5 ± 28.9 | | 101.8 ± 17.6 | | 104.9 ± 15.8 | | 112.8 ± 29.7 | 0.08 |
| CRP | | 2.4 (1.3-4.3) | | 3.0 (1.7-6.0) | | 3.1 (1.9-6.2) | | 6.3 (3.0-10.2) | <0.01 |
| **Sclerostin** | **Q1** | | **Q2** | | **Q3** | | **Q4** | |  |
| Age (years) | 58.8 ± 6.4 | | 57.5 ± 7.2 | | 57.7 ± 6.4 | | 57.8 ± 6.7 | | 0.75 |
| Women, n(%) | 50 (91) | | 45 (83) | | 42 (78) | | 41 (75) | | 0.13 |
| Diabetes duration (years) | 6.4 (3.3-10.3) | | 6.1 (2.7-11.2) | | 5.0 (1.5-9.1) | | 3.7 (2.2-7.7) | | 0.19 |
| History of CVD, n(%) | 10 (18) | | 7 (13) | | 8 (15) | | 5 (9) | | 0.57 |
| BMI (kg/m2) | 27.9 ± 4.9 | | 30.2 ± 5.1 | | 29.7 ± 4.3 | | 29.8 ± 4.9 | | 0.06 |
| Systolic BP (mmHg) | 142.3 ± 21.7 | | 141.3 ± 21.7 | | 141.0 ± 21.3 | | 142.3 ± 19.0 | | 0.98 |
| Current smoker, n(%) | 16 (30) | | 11 (20) | | 13 (24) | | 9 (16) | | 0.44 |
| Total cholesterol | 4.8 ± 1.0 | | 5.0 ± 0.9 | | 5.5 ± 1.3 | | 5.5 ± 1.2 | | 0.98 |
| HDL | 1.1 ± 0.3 | | 1.1 ± 0.3 | | 1.1 ± 0.3 | | 1.0 ± 0.3 | | 0.80 |
| HbA1c (%) | 7.8 ± 1.5 | | 7.6 ± 1.4 | | 8.4 ± 1.7 | | 8.4 ± 1.9 | | 0.03 |
| eGFR | 107.6 ± 27.3 | | 107.1 ± 18.4 | | 101.7 ± 16.3 | | 112.7 ± 30.6 | | 0.13 |
| CRP | 3.5 (1.0-5.7) | | 3.1 (1.6-6.6)0 | | 3.5 (2.2-7.1) | | 3.1 (2.0-6.5) | | 0.73 |
| Data are shown as frequencies (%), mean ± standard deviation or median (interquartile range). BMI = body mass index, eGFR = estimated glomerular filtration rate. | | | | | | | | | |


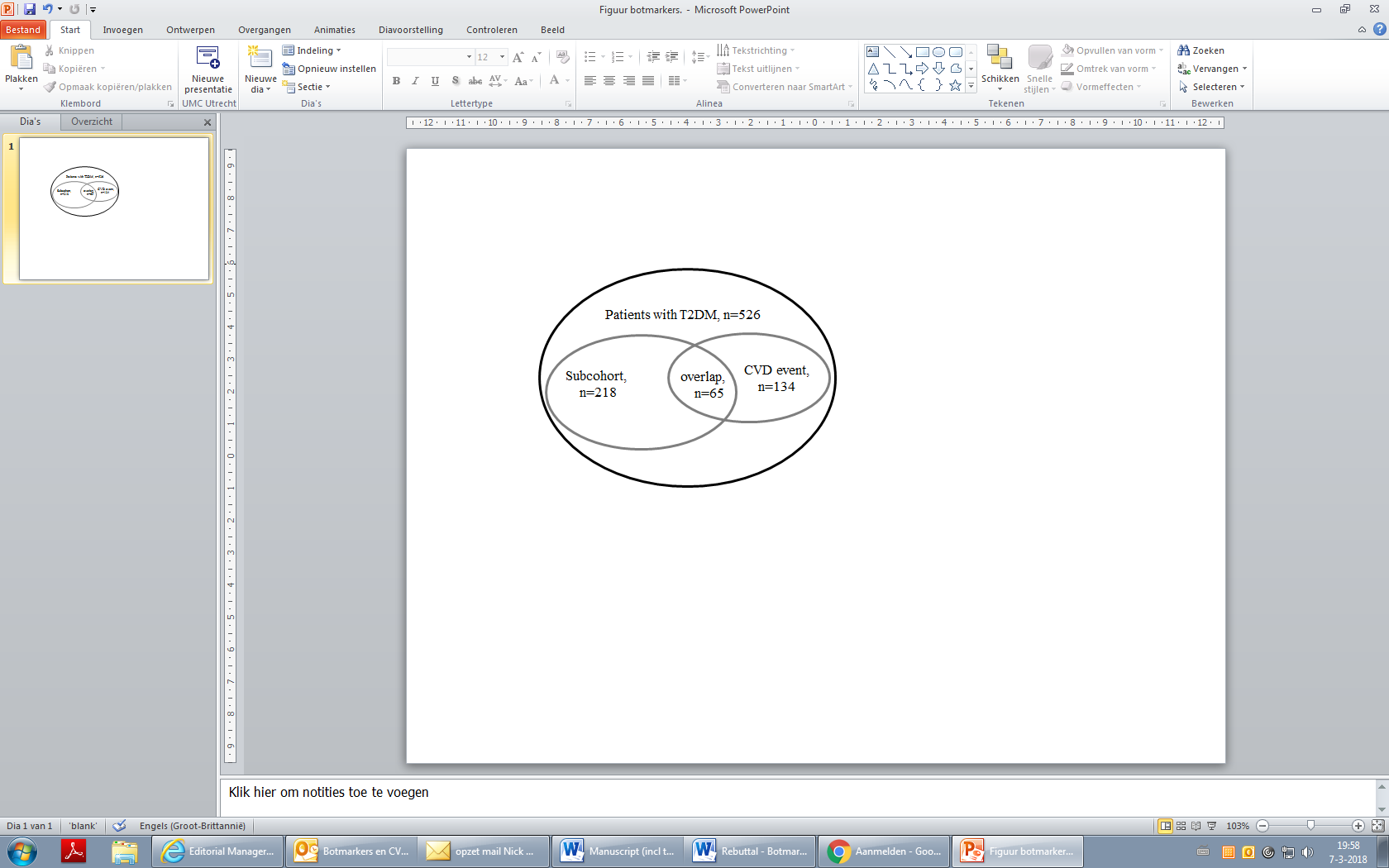


**Supplementary figure 1.** Case cohort sampling in the EPIC-NL cohort. (T2DM=type 2 diabetes mellitus, CVD=cardiovascular disease)
